# La tumeur desmoïde du mésentère: cause rare de masse abdominale chez la femme

**DOI:** 10.11604/pamj.2014.17.257.3292

**Published:** 2014-04-08

**Authors:** Majdouline Boujoual, Mariam Amrani, Abdesslam Bougtab

**Affiliations:** 1 Gynécologie Obstétrique, faculté de Médecine et de pharmacie Oujda, Maroc; 2 Anatomie pathologique, Institut National d'Oncologie Rabat, Maroc; 3 Chirurgie II, Institut National d'Oncologie Rabat, Maroc

**Keywords:** Tumeur desmoide du mésentère, imagerie, anatomie pathologique, chirurgie, Desmoid tumor of the mesentery, imagery, pathology, surgery

## Abstract

La tumeurdesmoïde du mésentèreest unetumeur fibreuse localement invasive, sans pouvoir métastatique, mais avec une tendance à la récidive. Du fait de sa rareté, ses circonstances de découverte très variées et son expression clinique aspécifique, elle pose un problème majeur de prise en charge thérapeutique. Nous rapportons un nouveaucas de tumeurdesmoïde du mésentère chez une femme de 35 ansprésentant un tableau de masse abdominale. La tomodensitométrieétait en faveur d'une tumeur solide du péritoine en contact intime avec une anse jéjunale. Unerésection - anastomosedu segment intestinal a été réalisée du fait du caractère envahissant de la tumeur. L'examen histologiqueconfirmé le diagnostic de tumeur desmoïde du mésentère infiltrant le méso grêlique sans atteinte de la paroi intestinale.

## Introduction

La tumeur desmoïde du mésentère est une fibromatose rare et profonde [1], ayant un aspect borderline entre le fibrome et le fibrosarcome. En effet, elle a une agressivité locale sans pouvoir métastatique mais avec une forte tendance à la récidive [2–4]. Elle est plus fréquente dans le syndrome de Gardner, le plus souvent asymptomatique, peut toutefois se révéler par des complications digestives, vasculaires ou urologiques [1]. Du fait de sa rareté, ses circonstances de découverte très variées et son expression clinique aspécifique, elle pose un problème majeur de prise en charge thérapeutique [5]. Nous rapportons un nouveau cas de tumeur desmoïde du mésentère colligé au service de Chirurgie II à l'Institut National d'Oncologie Rabat, nous insisterons, à travers une revue de littérature, sur les modalités diagnostiques, thérapeutiques et évolutives de ces tumeurs.

## Patient et observation

**Figure 1:**
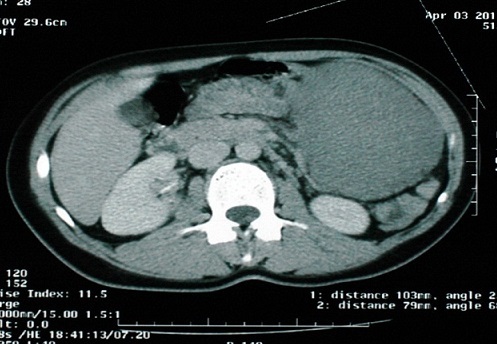
TDM abdominale montrant une masse arrondie tissulaire homogène, à contours réguliers de 103/79 mm entrant en contact intime avec une anse jéjunale tout en restant à distance de la queue du pancréas, reins, rate, utérus et des ovaries

**Figure 2:**
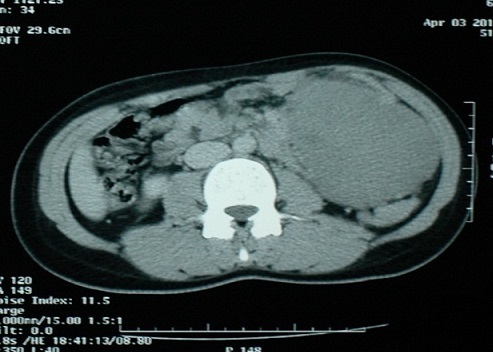
TDM abdominale montrant une masse arrondie tissulaire homogène, à contours réguliers de 103/79 mm entrant en contact intime avec une anse jéjunale tout en restant à distance de la queue du pancréas, reins, rate, utérus et des ovaries

Patiente âgée de 35 ans, sans antécédents pathologiques notables, présentant depuis un an une masse abdominale, augmentant progressivement de volume, avec sensation de pesanteur, sans signe urinaire ni digestif associé. Son examen abdominal a retrouvé une masse ferme, indolore, mobilisable s’étendant du flanc gauche à l'ombilic. Les touchers pelviens étaient sans particularités. L’échographie abdomino pelvienne a objectivé une masse intra péritonéale tissulaire grossièrement arrondie peu vascularisée, mesurant 102/ 77 mm, refoulant en arrière l'aorte, la veine cave inférieure et leurs branches iliaques. La TDM abdominale a montré la présence au niveau de la région paramédiane gauche de la cavité péritonéale d'une masse arrondie tissulaire homogène, à contours réguliers, mesurant 103/79 mm, entrant en contact intime avec une anse jéjunale tout en restant à distance de la queue du pancréas, reins, rate, utérus et des ovaires. Sa prise de contraste était modérée après injection de produit de contraste. Cet aspect évoquait une tumeur solide du péritoine (Figure 1, Figure 2).

**Figure 3:**
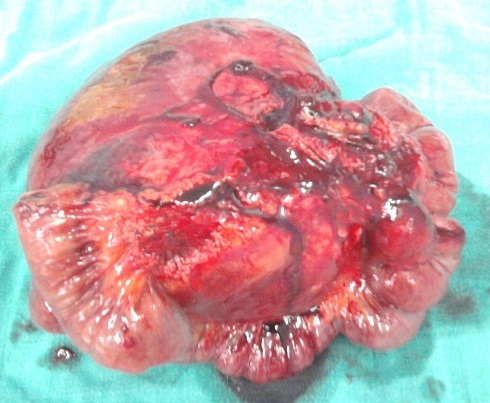
Aspect macroscopique de la tumeur

**Figure 4:**
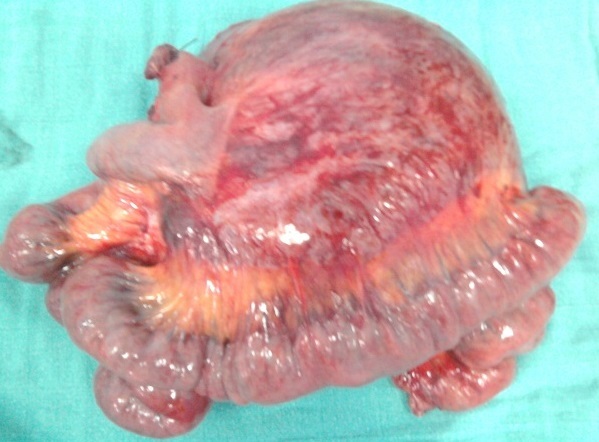
Aspect macroscopique de la tumeur

**Figure 5:**
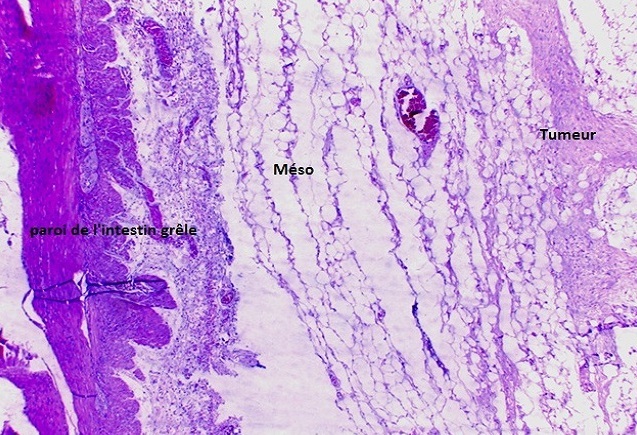
Tumeur desmoide du mésentère: jonction avec méso et paroi du grêle. Hématoxyline-eosine (HE)X 4

**Figure 6:**
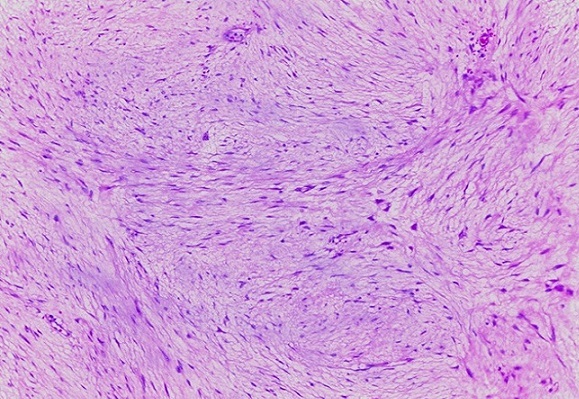
Tumeur desmoide du mésentère: aspect fasciculé. HE X10

**Figure 7:**
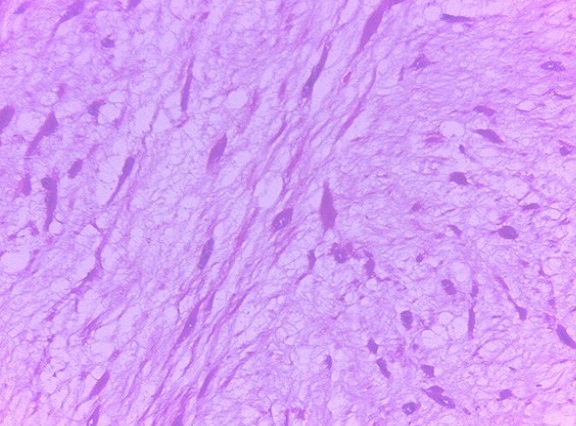
Tumeur desmoïde: aspect fasciculé. HE, GX40

La patiente a bénéficié d'une laparotomie exploratrice par une incision médiane sus et sous ombilicale qui a mis en évidence une masse solide intimement liée à l'intestin grêle avec envahissement par endroit, ce qui a nécessité une résection avec anastomose termino terminale de l'intestin grêle. La tumeur mesurait 19 x 15 x 7 cm, était appendue au fragment intestinal réséqué de 75 cm sans lésion macroscopique d'envahissement à l'ouverture de la pièce opératoire (Figure 3, Figure 4). Son analyse microscopique a mis en évidence une prolifération tumorale lâche et fasciculée de noyaux allongés à bouts effilés dans un cytoplasme éosinophile sans atypie ni mitose. La tumeur infiltrait le méso grêlique sans atteinte de la paroi intestinale. Cet aspect était évocateur d'une tumeur desmoïde du mésentère (Figure 5, Figure 6, Figure 7). Les suites post opératoires étaient simples, la patiente a été mise sous Tamoxifène à raison de 20 mg par jour, elle est toujours suivie en consultation sans récidive notable.

## Discussion

La tumeur desmoïde est rare représente moins de 0.03% de toutes les tumeurs et 3.5% des tumeurs fibreuses [5]. Elle peut se localiser soit dans les aponévroses des muscles périphériques (45% des cas), soit dans les muscles de la paroi abdominale (45% des cas) ou plus rarement dans les régions mésentériques ou rétropéritonéales (10% des cas) [6]. En effet, elle touche le plus souvent le mésentère de l'intestin grêle [5], rarement le mésentère iliocolique, le grand épiploon et le rétro péritoine [7, 6].

La plupart des tumeurs desmoides mésentériques sont sporadiques, 10% d'entre elles entrent dans le cadre du syndrome de Gardner [6], sont associées à la polypose colique familiale par mutation du gène APC [8]. Dans ce cas, ces tumeurs sont plus agressives, responsables de 11% des cas de décès [2, 5]. Son étiopathogénie n′est pas claire. Elle pourrait résulter de la sommation de trois facteurs: une initiation de la prolifération cellulaire, éventuellement suite à un traumatisme [9, 10]; un effet promoteur des stéroïdes sexuels: soutenu par leur prédominance chez la femme en activité génitale, la possibilité d'aggravation lors des grossesses et de régression spontanée à la ménopause, de même que la présence fréquente de récepteurs hormonaux expliquant sa possible hormono-sensibilité [4, 9, 11]; un terrain génétique particulier marqué par un trouble de la régulation de la croissance fibroblastique [9].

Elles sont plus fréquentes chez l'adulte jeune avec un pic de fréquence à l’âge de 30 ans et touchent aussi bien les hommes que les femmes [2, 6]. Leur mode de révélation le plus fréquent est une masse abdominale asymptomatique parfois douloureuse [5]. En effet, elles demeurent souvent asymptomatiques jusqu’à l'atteinte de taille importante [2, 8], entrainant des complications telles que: occlusion, ischémie ou perforation intestinale, hydronéphrose, rupture aortique, fistule digestive, thrombose veineuse profonde, compression neurologique et altération de la fonction iléale [5, 12].

Les caractéristiques échographique de la tumeur sont aspécifiques dépendent de sa teneur en collagène, en fibroblaste et de sa vascularisation [7]. C'est la TDM qui représente l'imagerie de première intention, elle permet de caractériser la tumeur, de guider les biopsies [2, 7] et d’établir des critères de mauvais pronostic notamment: un diamètre <; 10 cm, des localisations mésentériques multiples, l'enchâssement extensif de l'intestin grêle ou de l'artère mésentérique supérieure, l'hydronéphrose bilatérale [5]. Typiquement, elle se présente comme une massebien circonscrite, attachée àl′intestin grêle, parfois mal définieet irrégulière témoignant son caractère infiltrant. Ces données de la littérature concordent avec notre cas. L'IRM est surtout recommandée dans le suivi [2, 7], puisque sa meilleure résolution permet de différencier les remaniements post thérapeutiques, d'une récidive et de prévoir la croissance tumorale [5]. Son aspect est souvent hétérogène, en signal intermédiaire ou en hypo signal global en T1 et en signal mixte en T2 [13]. Quant à la colonoscopie, elle doit être réalisée de principe afin d′éliminer l′association rare mais possible avec une polypose colique [9].

Histologiquement: la lésion est mal limitée infiltrant les tissus adjacents. La prolifération est faite de cellules allongées, fusiformes, de petites tailles, avec un noyau de petite taille, sans atypies. Elle fixe la Vimentine et l'Actine musculaire [14].

L'exérèsechirurgicale large est letraitement de première intention des tumeurs desmoïdesmésentériques [2]. Toutefois, ces tumeurs posent le problème de résécabilité d'une part, par leur localisation intra mésentérique notamment leur contact avec les vaisseaux mésentériques [5], d'autre part par l'infiltration des organes de voisinage [14]. Par conséquent, la majoritédes casnécessitentune résectiondusegment intestinal atteint [2], d'autant plus que le risque de récidive élevé dans ces localisations (50% à 80% des cas) [5], dépend surtout de la qualité des marges d'exérèse (rechute de 27% si marge histologiquement saine contre 54% si marge envahie).

La fréquence des rechutes locales et la difficulté de prise en charge des formes évoluées ont conduit à la mise en route de traitements adjuvants [15]: la radiothérapie est indiquée en cas de résection incomplète, de tumeur inopérable ou de rechute. Toutefois, sa toxicité digestive, et son effet potentiellement carcinogène rendent son utilisation délicate [5, 11, 14]; le traitement anti-hormonal (Tamoxifène 20mg/ jour) seul ou en association avec un anti-inflammatoire non stéroïdien (l'Indométacine: 75mg/jour) permet de longues stabilisations et une réponse objective dans plus de 50% des cas. En cas d′échec ou de rechute, une seconde ligne d′hormonothérapie (torémifène, inhibiteurs de l′aromatase, dérivés de la gonadolibérine) peut être efficace; les polychimiothérapies sont volontiers proposées aux tumeurs rapidement évolutives, ou résistantes [11]. En effet, l'association de faibles doses de Méthotrexate et Vinblastine constitue un traitement efficace dans 70% des cas [5].

Le pronostic est bon après exérèse complète. Toutefois, en cas de récidivelocale, le traitement chirurgical itératif est grevé de morbi-mortalité importante.En effet, la forte infiltration des viscères abdominauxreprésente la causeultimede décèsaprès plusieurs années [7].

## Conclusion

La tumeur desmoïde du mésentère une prolifération fibreuse infiltrante qui pose au praticien: un problème diagnostique: elle peut mimer radiologiquement une tumeur maligne; un problème thérapeutique: du fait de son pouvoir infiltrant et sa proximité des vaisseaux mésentériques; un problème évolutif: par le risque de récidive locale justifiant une surveillance rapprochée.
